# Clinical Lectures and Essays

**Published:** 1876-01

**Authors:** 


*Clinical Lectures and Essays.* By SIR JAMES PAGET, Bart. Edited by HOWARD MARSH, F. R. C. S. New York: D. Appleton & Co., 1875. Buffalo: Herger and Ulbrich.

Most of these lectures and essays have appeared in Medical Journals or Hospital Reports, but in the present form they are more readily obtained by the general professional reader, and all who have read and admired these essays as they have appeared elsewhere will most heartily thank the author for publishing them in the present form.

The reader is at once pleased with the earnstness and pains-taking of the lecturer. He allows no detail, no matter how minute, to escape his attention which may in the end influence the termination of the case. No circumstance is considered too trival to engage his attention if thereby he may prevent his hearers from falling into error.

The author says: "I do not suppose that the book contains much, if anything, which is not known to those who are in large surgical practice, or familiar with surgical literature; but it is not intended for these. Its chief purpose will be attained if it be useful to the students, and to those who have too few opportunities of studying surgery in either large practice or books."

The subjects considered are the following: The various risks of operations, the calamities of surgery, stammering with other organs than that of speech, cases that bone-setters cure, strangulated hernia, chronic pyæmia, nervous mimicry, treatment of carbuncle, sexual hypochondriasis, gouty phlebitis residual abscess, dissection-poisons, quiet necrosis, senile scrofula, scarlet fever after operations, and notes for the study of some constitutional diseases.

These subjects are those which every physician encounters in his every day practice, and the reader may enquire if there is anything in these subjects which has not been said over and over by authors and teachers. The author admits that there may be nothing new in the work, but he has treated each subject in such a clear logical manner and withal in such honesty of purpose that the reader cannot help gathering new and valuable ideas on each subject.

When he has a doubt as to diagnosis or prognosis he freely admits it, thus showing to the young professional man that he is not the only one who some times doubt; when his cases terminate differently from what had been anticipated, he carefully scrutinizes each step in their progress, and does not hesitate to expose his own errors for the warning of his reader.

We wish that we had the time and space to go fully into the many valuable and excellent features of the work, but to do that would be to occupy our entire Journal, we can only say in conclusion that the work is as much adapted to those engaged in large surgical practice as to students and those unfamiliar with surgical practice or literature.

